# Alternative treatment for open bite Class III malocclusion in a child with Williams-Beuren syndrome

**DOI:** 10.1590/2176-9451.20.1.097-107.oar

**Published:** 2015

**Authors:** Giovanni Modesto Vieira, Eduardo Jacomino Franco, Denise Falcão Pinheiro da Rocha, Laudimar Alves de Oliveira, Rivadávio Fernandes Batista Amorim

**Affiliations:** 1 Professor, Postgraduate program in Orthodontics, Brazilian Dental Association (ABO-DF); 2 PhD in Health Sciences, University of Brasília (UnB); 3 Adjunct professor, Department of Dentistry, UnB; 4 Professor and head, PhD program in Medical Sciences, UnB

**Keywords:** Chromosome deletion, Open Bite, Angle Class III malocclusion treatment, Protraction of the maxilla

## Abstract

Williams-Beuren syndrome (WBS) is a rare genetic condition that affects approximately 1 in every 20,000 - 50,000 live births. WBS children have specific skeletal deformities, dental malformations and rare lingual muscle dysfunction. The need for orthodontic and orthognathic therapy has arisen and has been considered a real clinical challenge even for experienced professionals, once it requires a complex and individualized treatment plan. This study reports a case of orthopedic expansion of the maxilla, in which a modified facial mask was used for protraction of the maxillary complex associated with clockwise rotation of the maxilla. In addition, special considerations about treatment time and orthopedic outcomes are discussed.

## Abstract (pt)

A síndrome de Williams-Beuren (WBS) é uma doença genética rara, acometendo, aproximadamente, de 1:20.000 a 1:50.000 crianças nascidas. As crianças com WBS têm deformidades esqueléticas específicas, má formações dentárias e, algumas vezes, disfunção muscular da língua. As necessidades ortodônticas e ortognáticas têm sido consideradas um verdadeiro desafio clínico, até mesmo para aqueles profissionais com vasta experiência, uma vez que requerem um plano de tratamento individualizado e complexo. Esse relato de caso aborda uma expansão ortopédica da maxila, em que foi utilizada uma máscara facial modificada para protração do complexo maxilar, acompanhada de uma rotação horária da maxila. Além disso, considerações especiais sobre o tempo de tratamento e resultados ortopédicos são discutidas.

## INTRODUCTION

Williams-Beuren syndrome (WBS), OMIM #194050, was first described by Williams in 1961. In 1962, Beuren thoroughly described the major features that comprise the clinical phenotype of this condition.^1^^,^ 2 This rare genetic disorder is caused by hemizygous deletion of 1.5 to 1.8 Mb on 7q11.23,^3^ and has an autosomal dominant pattern, without gender predilection^2^^,^ 4 with an estimated prevalence of one case in every 7500 people.^5^

WBS patients are short in stature, have microcephaly, transient hypercalcemia, mental disability, social disinhibition, and cardiovascular abnormalities, such as supravalvular aortic stenosis.^1^^,^ 6 ^,^ 7 ^,^ 8 In addition, the syndrome is characterized by changes in the craniofacial skeleton other than microcephaly.

Cephalograms of 62 Caucasian individuals aged between 4.4 and 44.4 years old revealed that these patients present short base of the skull, flattening of the upper layer of parietal bone, as well as prominent occipital and frontal bones. Only a few cases of cleft palate have been reported in association with this syndrome.^9^ Another study conducted with 40 ten-year-old syndromic children found that 40.5% had agenesis of one or more than one permanent tooth, whereas 11.9% had agenesis of more than six permanent teeth associated with changes in mesiodistal and buccolingual dimensions.^10^

Moreover, it has been observed anterior inclination of the maxilla,^11^ accentuated inclination of the mandibular plane,^12^ and short base of the skull length.^11^^,^ 12 Furthermore, mentum deformities in combination with high mandibular plane angle also lead to retrusion of the mandible.^11^^,^ 13 Other functional aspects, such as mouth breathing, can also contribute to cause changes in the craniofacial complex.^14^ These characteristics hinder dental function and esthetics, thereby requiring complex orthodontic treatment.

Due to the significant oral and maxillofacial changes present in patients with WBS and the high relevance of clinical management in these cases, the purpose of this case report is to present an alternative approach to treat orthognathic malformation in a child with WBS. The procedure was performed in a two-stage treatment from 2002 to 2012.

## CASE REPORT

## Diagnosis, initial procedures and etiology

A mother of an eight-year-old boy sought dental care at an Orthodontics care unit, reporting that she had noticed malocclusion in her son. She reported no particular clinical event during pregnancy, no history of teratogenicity potential drug usage, and her son's birth as being at term. Furthermore, no cases of WBS were reported in her family medical history.

**Figure 1 -:**
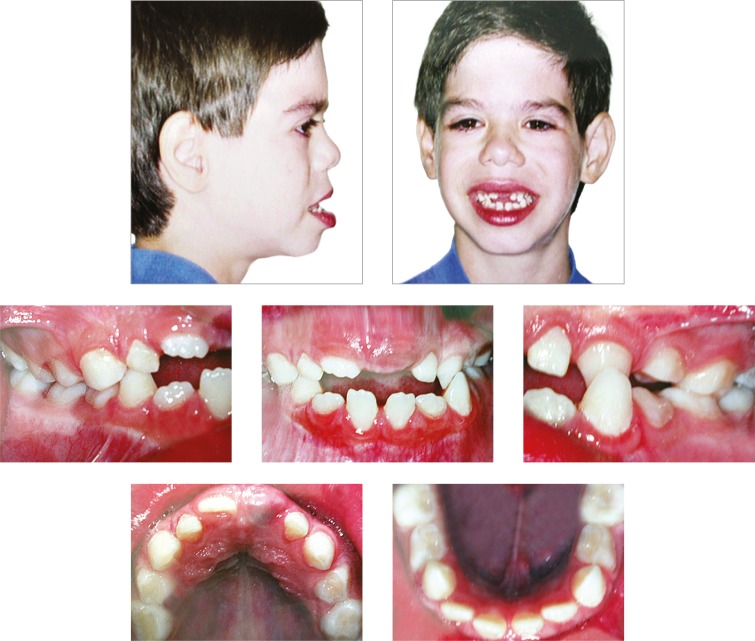
Photographs before treatment.

The boy had already been diagnosed with valvular aortic and pulmonary stenosis, as well as deficit in intellectual development. Additionally, he had a hoarse voice and sociable behavior. The patient had an "elfin face" appearance, with a small nose, long philtrum, prominent lips, and zygomatic flattening. Other signs included increased lower third of the face, large buccal corridor, nasolabial angle of 110^o^ and increased chin-neck line (Fig 1). With regard to the functional aspect, lip incompetence with mouth breathing and hypertrophied pharyngeal tonsils were observed. Intraoral examination revealed macroglossia with accentuated lingual interposition, small-sized teeth, generalized diastemas and good oral hygiene with absence of cavities or gingivitis.

**Figure 2 -:**
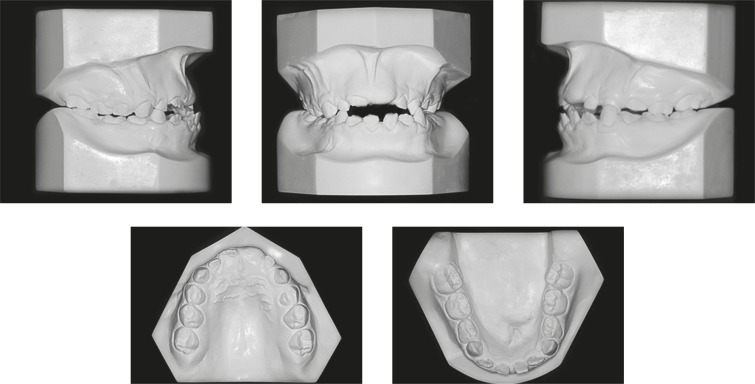
Photographs of dental casts before treatment.

**Figure 3 -:**
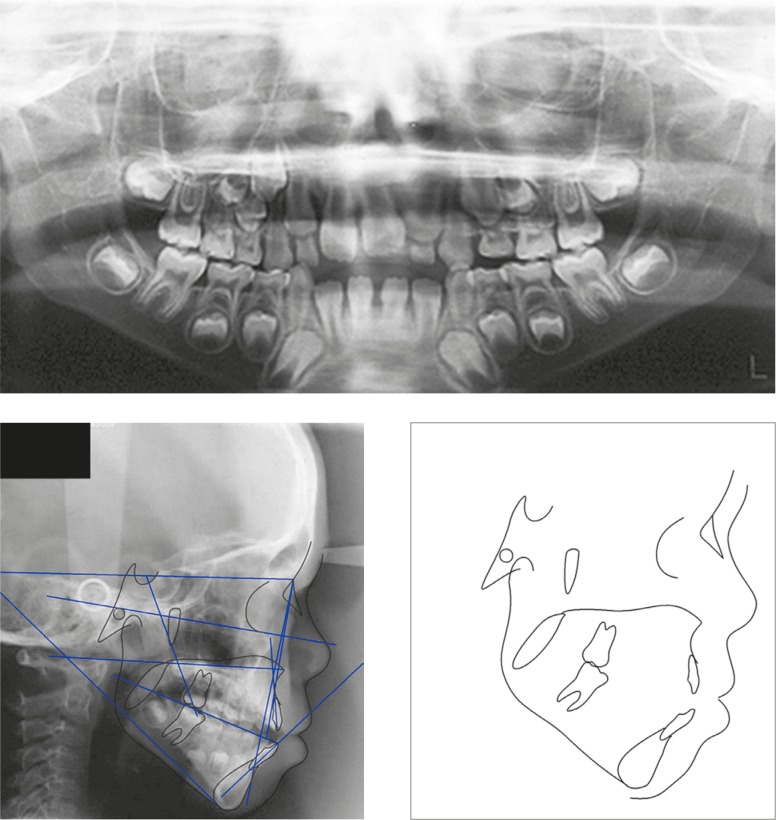
Panoramic radiograph and lateral cephalogram before treatment.

Initially, panoramic radiograph and teleradiography with lateral cephalograms were requested. They revealed early mixed dentition and corroborated the morphological characteristics of microdontia and tooth spacing. Moreover, agenesis of maxillary left first premolar and crossbite of left maxillary canine (Fig 2) were also evinced. The patient was diagnosed with Class III malocclusion, narrow maxilla, negative anterior overbite and overjet (overbite = -4 mm, overjet = -5 mm). Initial cephalometric analysis (Fig 3) revealed structural skeletal open bite, accentuated inclination of the gonial angle, counterclockwise rotation of the maxilla, increased lower anterior facial height (LAFH), negative overjet due to excessive protrusion of mandibular incisors, and slight retrusion of maxillary incisors (Table 1). Prognosis of facial growth was unfavorable due to clockwise rotation of the mandible associated with anterior-posterior maxillary deficiency.

## Treatment

**Table 1 -:** Cephalometric analysis - C0 treatment onset , 8 years old; C1: 11 years and 4 months old; C2: 12 years and 6 months old, C3: 15 years and 4 months old, C4 at the age of 17.

Measurement	Norm	C_O_	C_1_	C_2_	C_3_	C_4_
SNA (degrees)	82	80.84	84.97	85.48	87.2	85.2
SNB (degrees)	80	79.65	84.95	84.4	87.44	88.29
ANB (degrees)	2	1.2	0.02	1.07	-0.24	-3.09
SN.GO.GN (degrees)	32	40.53	33.87	34.44	32.51	34.94
MM (degrees)	28	40.9	37.74	37.17	35.37	38.63
SN.GN (degrees)	67	68.62	62.86	64.12	62.93	63.27
IMPA (degrees)	87	92.41	84.3	79.01	84.4	75.97
1/PLMx (degrees)	110	97.42	116.68	111.37	118.49	119.66
1.NA (degrees)	22	14.78	32.41	26.34	31.51	35.72
1.NB (degrees)	25	34.75	26.29	20.15	27	21.63
1-NA (mm)	4	5.25	6.9	6.2	7.48	9.88
1-NB (mm)	4	8.4	6.26	4.41	7.14	4.11
1.1 (degrees)	131	129.27	121.28	132.44	121.73	125.75
ANFH (mm)	__	-0.87	2.68	2.12	3.54	-0.32
PNFH (mm)	4	-6.17	4.3	0.77	7.18	5.24
Co-A (mm)	__	74.09	87	88.31	95.16	81.4
Co-GN (mm)	__	99.77	118.16	118.27	133.25	124.58
ALFH (mm)	__	69.57	70.34	70.64	77.22	75.15
NLA (degrees)	95 a 110	110.12	101.69	101.69	97.1	101.55

The goals of initial treatment were to restore muscle tone with competent lips and achieve appropriate lingual resting posture. Secondary objectives were to correct anterior open bite, attain adequate overjet, correct Class III molar relationship and achieve orthodontic alignment and leveling. Treatment planning included potential dental implant placement in the region of maxillary left first premolar after complete growth.

Orthodontic-orthognathic treatment was planned. Nevertheless, the patient's family refused it and chose to follow an orthopedic-orthodontic approach. Treatment was planned to be performed in two stages: orthopedic therapy to correct the transverse skeletal relationship and to improve sagittal skeletal relationship (anterior-posterior), followed by compensatory orthodontic treatment to correct dental vertical and sagittal discrepancies.

**Figure 4 -:**
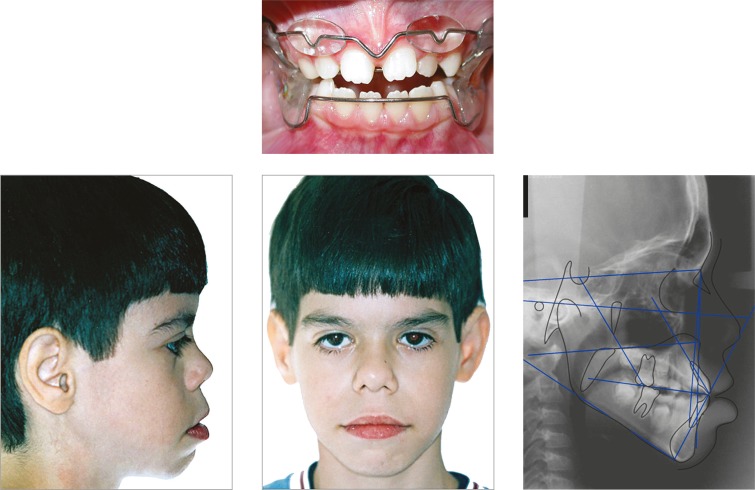
Completion of interceptive treatment with Frankel III before the use of facial mask. Note improvements in lip competence and muscle tone. Panoramic radiograph and lateral cephalogram after interceptive treatment.

Rapid maxillary expansion was performed at the age of 8 by means of a McNamara expander in combination with a vertical chin cup used at night. At the age of 9, the patient used a Frankel III appliance (Fig 4). There were clinical improvements in tongue position, which exhibited a tendency to rest on the lower anterior mandibular region. After 3 years of treatment, new rapid maxillary expansion procedures were carried out.

**Figure 5 -:**
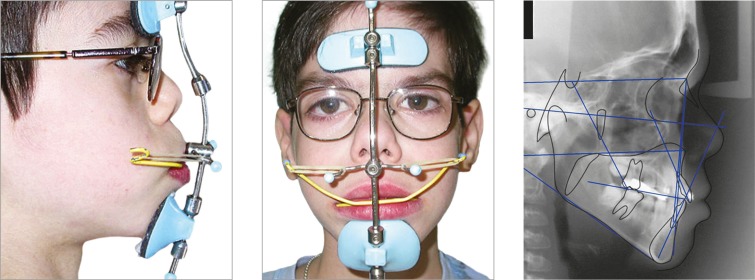
Nanda-modified facial mask with force application at the center of resistance in the maxillary complex. Lateral cephalogram after treatment with facial mask.

A Nanda-modified protraction headgear^15^^,^ 16 with facial mask was used to restore overjet and maxillomandibular relationship. The maxillary left canine, which was transposed, was mesialized by means of a 2 x 2 fixed appliance used as a guide, and facial mask with reverse traction used as anchorage (Fig 5).

**Figure 6 -:**
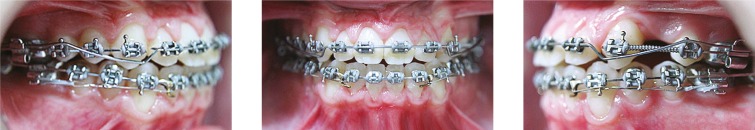
Patient at the age of 17 with extrusion maxillary arch in 0.019 x 0.025-in stainless steel arch wires.

At the age of 12, the patient was subject to follow-up so as to have growth and bucco-maxillofacial development monitored, and remained without further interventions for 3 years. At the age of 15, the patient presented with anterior crossbite relapse with excessive mandibular growth unaccompanied by maxillary compensatory growth. It was decided that the patient should, once again, undergo rapid maxillary expansion and maxillary protraction by means of the Nanda-modified facial mask^15^^,^ 16 used for approximately six months. Moreover, corrective treatment with straight-wire appliance was performed with the purpose of closing interincisal diastemas, as well as achieving dentoalveolar compensation and proper axial inclination of maxillary teeth. Dental alignment and leveling were accomplished with the use of light arch wires, and finished with ideal 0019 x 0025-in stainless steel arch wires and Class III elastics. A Connecticut intrusion arch adapted upside down in 0019 x 0025-in stainless steel sectional arch wires was used to correct residual anterior open bite (Fig 6).

**Figure 7 -:**
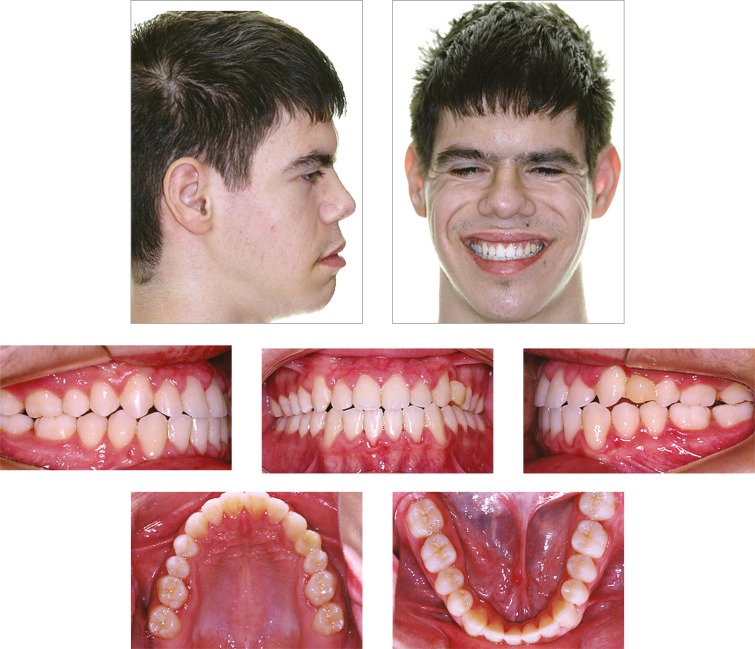
Photographs after treatment.

The orthopedic-orthodontic approach resulted in Angle Class I molar relationship, correction of anterior open bite, as well as adequate overjet and bilateral Class I canine relationship (Figs 7 and8). Alignment and leveling were achieved. Cephalometric analysis revealed a reasonable increase in the growth of the mandibular ramus with satisfactory dentoalveolar compensation and improved facial esthetics at the end of the treatment (Figs 9 and10).

**Figure 8 -:**
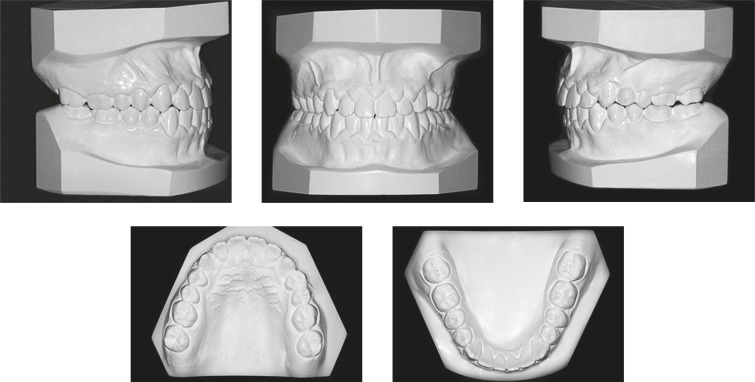
Dental casts after treatment.

**Figure 9 -:**
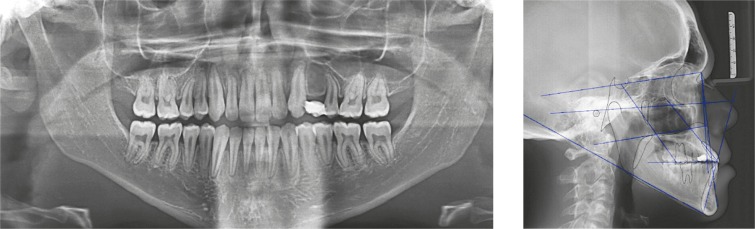
Panoramic radiograph and lateral cephalogram after treatment.

**Figure 10 -:**
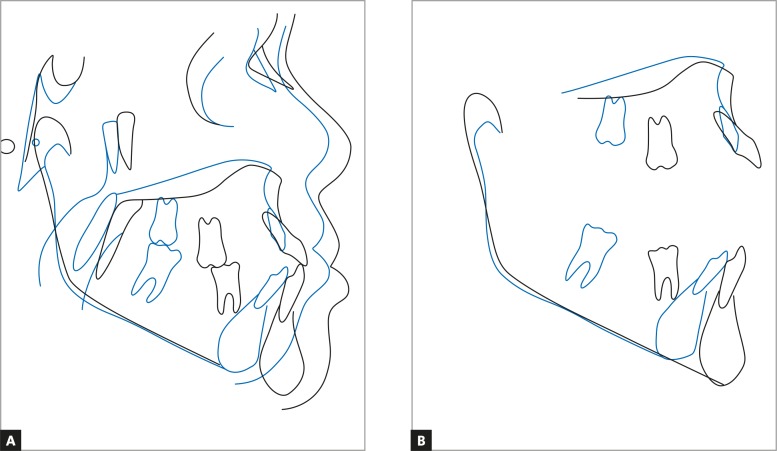
A) Cephalometric superimposition before and after treatment. B) Maxillary and mandibular superimposition.

## DISCUSSION

WBS requires complex and individualized treatment planning due to its specific skeletal deformities, dental malformations and lingual muscle dysfunctions. For this reason, it becomes a real clinical challenge, even for highly experienced professionals.^17^ "Elfin-like" or "gnome" appearance^18^ is very characteristic of this syndrome,^6^ and so it is periorbital swelling, large cheeks, flattened nasal bridge, relatively large mouth with prominent lips, long philtrum, flattening of zygomatic bones, small palpebral fissures, craniofacial asymmetry and depression of temporal bones.^11^ With regard to the peculiar facial characteristics of WBS, facial asymmetry was not the only one found in the present case.

As for intraoral and orthognatic characteristics, syndromic patients might present with microdontia, generalized diastemas, anodontia, caries, enamel hypoplasia, dental malocclusion, atypical deglutition and counterclockwise rotation of the maxilla accompanied by retruded mandible.^8^ Nevertheless, the present case presented with differential features, including Class III malocclusion associated with skeletal open bite and dental agenesis. Furthermore, there were no signs of structural or pathological defects in patient's teeth, except for agenesis and, thus, diastemas.

Short stature and early pubertal growth spurt are usually associated with the syndrome,^11^^,^ 19 ^,^ 20 ^,^ 21 and affect orthodontic treatment.^21^ Herzberg et al^22^ report that 31.8% of syndromic patients show Class II malocclusion, while 9.1% have Class III malocclusion. The present clinical case presented a patient with Class III malocclusion. However, a delayed pubertal growth spurt was present in disagreement with the literature in this regard. Gorlin et al^13^ reported that, in these patients, the mandibular arch is normally smaller in comparison to the maxillary arch, and the base of the skull is short despite maintaining normal angulation. Cephalometric analysis of our patient revealed counterclockwise inclination of the maxillary base, high mandibular plane angle with deficient mandible and chin bone.^11^

Despite deletion in elastin gene of patients with WBS, a factor that could be associated with potential deficiency in elastic-fiber formation of the periodontal ligament,^10^^,^ 23 ^,^ 24 no changes were found with regard to induced tooth movement or orthodontic movement relapse.

The greatest challenge of the present study was of functional nature, particularly with regard to motor impairment of facial skeletal muscles, muscular hypotonicity, and severe lingual interposition associated with significant macroglossia. Furthermore, the association between structural skeletal open bite with anterior crossbite led us to predict the potential need for orthognathic surgery at the end of the growth period.

Importantly, the child's mother was reluctant to accept any surgical treatment in the first moment. Thus, the patient underwent compensatory orthodontic-orthopedic treatment. The literature states that the best moment for maxillary protraction is between the ages of 8 and 9 years old due to maximization of orthopedic effects coinciding with the eruption of maxillary incisors^25^^,^ 26 ^,^ 27 and greater stability in subjects treated with facial mask.^28^ However, the patient had difficulty in using the facial mask due to lack of cooperation as a result of delayed cognitive (mental) maturity. Hence, initial treatment focused on normalization of functional aspects, particularly with regard to muscle hypotonicity and lingual muscles, thereby postponing maxillary protraction.

Interestingly, some studies have shown no differences in terms of orthodontic and orthopedic effects of maxillary advancement in patients in pre-pubertal growth spurt and pubertal growth peak, but there is a decrease in skeletal maxillary advancement (orthopedic) in subjects who initiated treatment of maxillary protraction after pubertal growth spurt.^20^ Subjects affected by WBS commonly present with advanced bone maturation.^19^^,^ 20 In the present case, the patient was in pre-pubertal growth spurt at the age of 11 at maxillary protraction therapy onset, which does not corroborate common clinical findings.^19^^,^ 20 This factor favored response to maxillary protraction treatment, however, it contributed to relapse occurring at the age of 15, one year and a half after pubertal growth spurt. The latter is in agreement with studies that found greater stability in cases treated with facial mask therapy^28^ at an early age.

Braun et al^29^ reported that maxillary protraction force applied at the oral commissure level and distant from the center of resistance of the maxillary complex would cause counterclockwise rotation of the maxilla (Fig 6) and increased lower anterior facial height,^29^ which would be unacceptable for this patient in particular. Nanda and Burstone^30^ also questioned traditional protraction force systems, since most cases of Class III malocclusion/molar relationship do not present deep overbite,^30^ in which case a pseudo-correction of Class III sagittal relationship occurs and probably leads to recurrence.

The Nanda-modified maxillary protraction facial mask^16^ consists of a modified extra oral arch^15^ with the insertion axis from distal to mesial direction.^16^ With this modified extra oral appliance, one may increase the outer arm of the appliance. Hence, force application passes through the center of resistance of the maxilla,^16^^,^ 31 thereby inducing a translational movement of the maxillary complex from posterior to anterior direction.^29^ The inclination of the outer arm can also be angled so that force application passes above the center of resistance of the maxilla and causes clockwise rotation of the maxilla without extrusion of its posterior region. Additionally, it improves incisor-lip relationship without undesirable mandibular rotation.^29^ This technique highly benefited our patient, as it avoided an increase in anterior facial height caused by undesirable clockwise rotation and also an increase in mandibular anterior open bite.

Rotational movements of the maxilla are important as they affect mandibular position. In patients with normal overbite and normal vertical skeletal relationships, anterior maxillary translation must be obtained by rotational-free moments.^31^^,^ 32 However, in patients with anterior open bite, as in this case report, movement of the maxilla must be accompanied by clockwise rotation, favoring counterclockwise self-rotation of the mandible. Both movements favor closure of anterior open bite and prevent increase in anterior vertical dimension which would be a negative issue for this case.^31^

## CONCLUSION

Although surgical-orthodontic treatment is commonly recommended for severe skeletal changes associated with WBS, depending on the degree of patient and family cooperation as well as the direction and magnitude of facial growth, there are alternative ways to manage syndromic patients effectively. Compensatory resources of orthodontic-orthopedic nature can be used to correct potential discrepancies, restore balance of occlusal relationship and improve esthetics and facial harmony.
